# Solid-State Nanopore-Based Nanosystem for Registration of Enzymatic Activity of a Single Molecule of Cytochrome P450 BM3

**DOI:** 10.3390/ijms251910864

**Published:** 2024-10-09

**Authors:** Yuri D. Ivanov, Angelina V. Vinogradova, Ekaterina D. Nevedrova, Alexander N. Ableev, Andrey F. Kozlov, Ivan D. Shumov, Vadim S. Ziborov, Oleg N. Afonin, Nikita V. Vaulin, Denis V. Lebedev, Anton S. Bukatin, Polina K. Afonicheva, Ivan S. Mukhin, Sergey A. Usanov, Alexander I. Archakov

**Affiliations:** 1 Institute of Biomedical Chemistry, 10, Pogodinskaya St., 119121 Moscow, Russia; angeluna1234@bk.ru (A.V.V.); nevedrova.kat@yandex.ru (E.D.N.); ableev@mail.ru (A.N.A.); afkozlow@mail.ru (A.F.K.); shum230988@mail.ru (I.D.S.); ziborov.vs@yandex.ru (V.S.Z.); sunweb@mail.ru (O.N.A.); alexander.archakov@ibmc.msk.ru (A.I.A.); 2 Joint Institute for High Temperatures of Russian Academy of Sciences, 13 Bd.2, Izhorskaya St., 125412 Moscow, Russia; 3 Laboratory of Renewable Energy Sources, St. Petersburg Academic University, 8/3, Khlopina St., 194021 St. Petersburg, Russia; nikitavaylin@mail.ru (N.V.V.); denis.v.lebedev@gmail.com (D.V.L.); antbuk.fiztek@gmail.com (A.S.B.); imukhin@yandex.ru (I.S.M.); 4 Institute for Analytical Instrumentation RAS, 31-33 Lit. A, Ivana Chernykh St., 198095 St. Petersburg, Russia; polina.afonicheva@gmail.com; 5 Institute of Chemistry, Saint Petersburg State University, 7/9, Universitetskaya Nab., 199034 St. Petersburg, Russia; 6 Higher School of Engineering Physics, Peter the Great Polytechnic University, 26, Polytehnicheskaya St., 194021 St. Petersburg, Russia; 7 Institute of Bioorganic Chemistry, National Academy of Sciences of Belarus, 5/2, Kuprevich St., 220141 Minsk, Belarus; usanov1948@inbox.ru

**Keywords:** nanopore detector, solid-state nanopore, cytochrome P450 BM3, enzyme functioning

## Abstract

Experimental methods of single-molecule enzymology allow scientists to determine physicochemical properties of distinct single molecules of various enzymes and to perform direct monitoring of functioning of enzymes at different steps of their catalytic cycle. The approach based on the use of solid-state nanopores is a promising tool for studying the functioning of single-enzyme molecules. Herein, this approach is employed for monitoring the functioning of cytochrome P450 BM3, which represents a very convenient model of cytochrome P450-containing monooxygenase systems. A nanopore of ~5 nm in diameter has been formed in a 40 nm-thick silicon nitride chip by electron beam drilling (EBD), and a single molecule of the BM3 enzyme has been entrapped in the pore. The functioning of the enzyme molecule has been monitored by recording the time dependence of the ion current through the nanopore during the reaction of laurate hydroxylation. In our experiments, the enzyme molecule has been found to be active for 1500 s. The results of our research can be further used in the development of highly sensitive detectors for single-molecule studies in enzymology.

## 1. Introduction

The activity of an enzyme against its substrate is a key characteristic parameter of any enzyme system [1]. The physical principles underlying the majority of methods, which are routinely employed in enzymology (and, moreover, in protein research in general), are based on the acquisition of signals from a large ensemble of enzyme molecules. Accordingly, the enzymatic activity measured by these methods represents a value which is averaged over the above-mentioned molecular ensemble [2]. At that, Ivanov et al. previously demonstrated that, in one and the same sample, physicochemical properties of different molecules of one and the same enzyme differ considerably [3]. This was achieved by using the so-called molecular detectors, which allow researchers to focus on individual enzyme molecules [4].

Currently, determination of physicochemical properties of single-enzyme molecules has become one of priority directions of modern biochemistry. Single-molecule enzymatic studies have been called single-molecule enzymology [5]. The key beneficial feature of this approach consists of direct determination of physicochemical properties of distinct single-enzyme macromolecules; these data are not averaged over a large ensemble of macromolecules—in contrast to macroscopic methods [3]. Methods employed in single-molecule enzymology allow researchers to perform direct monitoring of the functioning of enzymes at different steps of their catalytic cycle, characterizing their intermediate states [6].

Manipulations with single molecules of proteins and enzymes allow one to directly determine their structure, functional response, and to measure molecular forces [7,8,9].

Atomic force microscopy (AFM) was proven to be one of powerful methods for visualization and determination of physicochemical properties of single molecules of proteins [10] (including enzymes [7,8,9,10,11]) and their macromolecular complexes [12,13]. In AFM, single molecules are immobilized on a solid substrate and can be investigated under near-native conditions [13]. Sub-nanometer height resolution, provided by AFM in a semi-contact mode, allows one to precisely determine the heights of substrate-immobilized protein molecules, while the action of the AFM probe on them is kept to minimum. This allows AFM to be employed for the visualization of proteins and enzymes, the identification of their oligomeric state by height [11], and for monitoring the conformational dynamics of an enzyme in the course of its functioning [7]. These advantages of AFM were clearly demonstrated with the example of cytochrome P450 BM3 (hereinafter referred to as BM3) [3]—an enzyme of bacterial origin which catalyzes the monooxygenation of fatty acids [14,15,16]. The disadvantage of AFM consists of the impact of the AFM probe on the enzyme structure. This often leads to the deformation of the studied enzyme molecule, and this is why the height of the enzyme molecules measured by AFM can be underestimated [17]. Accordingly, the enzyme’s activity can change. In the case of nanopore-based detection used in our present study, this limitation is removed, since there is no impact of the AFM probe on the enzyme. This allows one to study the activity of an enzyme when distortion of its structure by the AFM probe is critically important.

Currently, an alternative nanopore-based approach to studying the functioning of single-enzyme molecules is being developed [18]. This nanopore-based approach does not require immobilization of the studied enzyme, thus being devoid of the above-mentioned disadvantage of AFM. In this approach, a nanopore with a size comparable with that of the enzyme is formed in a diaphragm, and the enzyme under study is confined in the nanopore. The diaphragm is placed between two chambers (called cis- and trans-, respectively) which are filled with an electrolyte solution. Application of voltage to the chambers separated by the nanopore with the confined enzyme molecule induces an ion current through the nanopore. Functioning of the enzyme leads to fluctuations in the measured ion current [19,20,21,22,23,24].

Biological (natural) nanopores are often used for this purpose [23]. These nanopores are obtained with the use of pore-forming proteins such as α-hemolysin or cytolysin A [18,23]. Biological nanopores have two significant drawbacks. The first one is the pore’s limited size, which is determined by the structure of the pore-forming protein [18]. This factor significantly limits the list of enzymes available for studying with the use of biological nanopores. The second drawback of biological nanopores is their limited chemical and time stability [18].

As an alternative to biological nanopores, artificial solid-state nanopores were proposed for single-molecule studies of proteins [18,25,26,27]. These nanopores can be formed in silicon nitride (Si_3_N_4_, further referred to as SiN) or silicon dioxide [28] membranes with a commercially available electron microscope [29,30]. This method was called electron beam drilling (EBD) [29,31]. The EBD method is based on standard silicon technology [29]. Accordingly, EBD is versatile and relatively simple, allowing one to fabricate nanopore-based devices for both biomedical research [18,32] and quantum electronics [30].

The use of solid-state nanopores allows for monitoring of the activity of single-enzyme molecules against their substrate, as was recently demonstrated with the example of horseradish peroxidase (HRP) [33]. HRP is a widely employed model enzyme with well-known properties, thus providing the correct interpretation of data obtained throughout studying it with novel methods, including nanopore-based determination of enzymatic activity [33]. These novel methods, tested with HRP, should subsequently be employed for the investigation of other enzyme systems, and this is what we have carried out in the present research with respect to BM3.

Herein, a solid-state nanopore has been employed for the registration of activity of a single molecule of BM3. This enzyme pertains to a heme-containing superfamily of cytochromes P450 which play important role in metabolic pathways. BM3 represents a self-sufficient enzyme whose functioning does not require the presence of additional partner proteins in the system. In the structure of BM3, the two domains—the reductase one and the heme one—are joined in a single polypeptide chain [15,34,35]. BM3 catalyzes the hydroxylation of laurate, producing almost exclusively 11-hydroxylaurate [36]. In general, cytochromes P450, related to the ω-hydroxylation of fatty acids, are widely scattered phylogenetically, while metabolites produced in such reactions play important structural and physiological roles [36]. This feature of BM3 explains its use as a very convenient simple model of cytochrome P450-containing monooxygenase systems. With respect to a nanopore-based enzymatic activity study, this feature of BM3 has also allowed us to simplify the experiment workflow.

Of course, the nanopore-based approach has certain limitations, which are related to the size of the enzyme of interest. Firstly, if the size of the nanopore is larger than that of the enzyme, the latter can pass through the nanopore, making the registration of its activity very difficult. The second limitation is related to the enzyme structure. Namely, if this structure is insufficiently rigid, the enzyme can slip through the nanopore under the influence of the electric field. This will also hinder the monitoring of the enzyme activity. Furthermore, it should be kept in mind that entrapment in a nanopore can seriously affect enzymes with a labile structure. Another crucial point is providing the availability of the enzyme’s active site to the substrate. The active site of a nanopore-confined enzyme can become blocked and unavailable to the substrate. Careful selection of the nanopore size can help in this respect.

In our experiments, we have found that the use of an artificial nanopore formed in a SiN structure has allowed us to register the functioning of a single BM3 molecule. The time dependence of the ion current through the nanopore with an entrapped BM3 molecule has been recorded in the course of its functioning.

## 2. Results

According to the literature, the size of the BM3 molecule makes up 12.9 × 7 × 6 nm [37]. At the 10^−8^ M concentration used in our experiments, BM3 exists in dimeric form [15]. Accordingly, the enzyme did not pass through the 5 nm nanopore from the cis-chamber to the trans-chamber.

**Figure 1:**
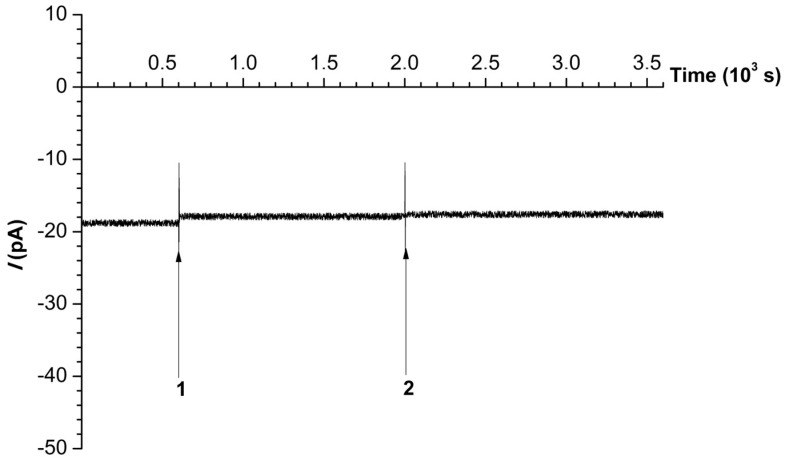
Typical time dependence of the ion current through the nanopore formed in a 40 nm-thick SiN chip recorded in the control experiment in the absence of the enzyme at a −400 mV voltage in 2 mM PBS-D. Numbers indicate the time points of the addition of 500 µM sodium laurate (1) and 200 µM NADPH (2).

In the control experiments performed in absence of the enzyme, a 0.5 mM solution of sodium laurate and, afterwards, a 0.2 mM solution of NADPH were added to the buffer in the cis-chamber. These additions did not cause any changes in the ion current through the nanopore, as shown in Figure 1.

**Figure 2:**
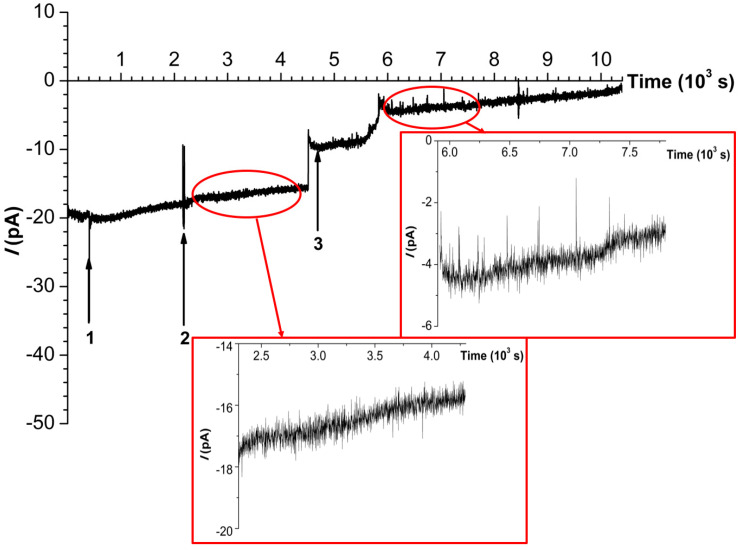
The typical time dependence of ion current through the nanopore (*I*(*t*) dependence) formed in a 40 nm-thick SiN chip recorded in the working experiment at a −400 mV voltage in 2 mM PBS-D. Numbers indicate the time points of the addition of the enzyme (1), 500 µM sodium laurate (2), and 200 µM NADPH (3). Insets in red square frames show enlarged fragments of the *I*(*t*) dependence.

In working experiments, 10 nM solution of BM3 in 2 mM PBS-D was added to the cis-chamber. The typical time dependence recorded in the working experiment is shown in Figure 2.

An enzyme molecule was entrapped in the nanopore after the addition of the enzyme to the cis-chamber of the measuring cell (see Figure 2, time point #1, ~400 s). At that point, the noise observed after enzyme entrapment was ~1 pA. After the entrapment of a single BM3 molecule in the nanopore, we investigated the activity of this molecule in the reaction of laurate hydroxylation. This has been performed by monitoring the time dependence of the ion current (*I*(*t*) dependence) through the nanopore with the entrapped enzyme molecule. With this purpose, firstly, a 500 µM solution of sodium laurate was added to the cis-chamber (see Figure 2, time point #2, 2200 s). At that point, a (−400) mV voltage was applied to the cis-chamber. Secondly, a 200 µM solution of the electron donor NADPH was added (see Figure 2, time point #3, ~4800 s).

As seen in Figure 2, under the conditions of the working experiment, the initial ion current through the nanopore before the addition of the enzyme was *I* ≈ (−20) pA. After the enzyme addition, *I* did not change considerably, though it exhibited a slight tendency to increase. The left inset in Figure 2 displays an enlarged fragment of the *I*(*t*) dependence between the 2300 s and 4400 s time points. After the addition of sodium laurate at ~2200 s (time point #2), this tendency remained the same until ~4600 s, when *I* abruptly decreased to (−8) pA without a subsequent increase until the addition of 200 µM NADPH solution at ~4800 s (time point #3). The addition of NADPH initiated the reaction of laurate hydroxylation by the nanopore-entrapped BM3 molecule. Additionally, the fragment of the *I*(*t*) dependence shown in Figure 2 (right inset) clearly indicates numerous fluctuations in the ion current, which started at ~6000 s and continued to occur until ~7500 s. The amplitude of these fluctuations exceeded 2 pA, reaching 3 pA. Accordingly, the nanopore-entrapped enzyme molecule was active for ~1500 s.

## 3. Discussion

We have investigated the functioning of a single molecule of cytochrome P450 BM3 entrapped in a solid-state nanopore. The latter has been formed in a 40 nm-thick SiN chip by EBD. The time dependencies of the ion current flowing through the nanopore in different situations have been recorded and analyzed. Entrapment of the BM3 molecule has not led to any significant changes in the ion current—neither has the addition of laurate to the cis-chamber. However, the addition of NADPH has induced clearly distinguishable ionic current pulses. These pulses correspond to changes in the geometry of the nanopore lumen, which is partially blocked by the enzyme molecule. The changes in the nanopore lumen correspond to fluctuations of the enzyme molecule during its functioning. Namely, the addition of NADPH at ~4800 s did not cause the immediate occurrence of any considerable fluctuations in the ion current. These fluctuations only occurred after 6000 s. The observed *I*(*t*) dependence can be explained by a shift of the enzyme molecule inside the nanopore during functioning. This shift has obviously led to the partial blockade of the nanopore’s lumen. The lumen blockade has, in turn, led to an increase in the intensity of the ion current peaks, which accompanied the functioning of the entrapped enzyme. In our experiments, the enzyme molecule has been found to be catalytically active for 1500 s.

Previously, nanopore-based detectors were successfully demonstrated to be applicable for the registration of single-enzyme molecules with the example of horseradish peroxidase (HRP). Tan et al. [27] employed a nanopore of a relatively large (>20 nm) diameter. The detector based on such a large nanopore was shown to allow the authors to detect HRP molecules. Since HRP is prone to aggregation in aqueous solutions [11,38], the ion current peaks registered by Tan et al. might be attributed to HRP aggregates or to several closely adjacent HRP monomers passing through the large nanopore whose size was an order of magnitude larger than the HRP molecule [11]. In our previous paper, we reported the results of experiments in which a single HRP molecule was entrapped in a much smaller (~5 nm) solid-state nanopore [33]. The use of this small nanopore allowed us to observe the functioning of the single HRP molecule [33]. Namely, the characteristic ion current pulses, accompanying the functioning of the HRP molecule, were registered [33]. In the present study, such a small (~5 nm) solid-state nanopore has been successfully employed for the registration of activity of single molecule of another enzyme (cytochrome P450 BM3) in the course of the catalysis of laurate in the presence of NADPH. It is to be noted that the *I*(*t*) dependence observed with cytochrome P450 BM3 was more complex than that in the case with HRP. Namely, we have observed a partial blockade of the nanopore by the nanopore-entrapped cytochrome during functioning as opposed to the previously reported case with HRP [33]. This fact is quite important, indicating the need for future in-deep studies of the functioning of nanopore-entrapped enzymes.

One of the most important characteristics of an enzyme system is the heterogeneity of the enzyme [39]. Namely, individual enzyme molecules in one and the same sample can function differently under the same conditions. Our present work is a pioneer study of functioning of a single cytochrome P450 BM3 molecule. In the future, we intend to conduct experiments on the determination of activity of different BM3 molecules.

The use of nanopores of different sizes can allow one to study the activity of enzymes of various types. It is important to emphasize that the EBD method of nanopore fabrication allows one to precisely tune the size of solid-state nanopores [29]. Indeed, the major advantage of single-enzyme technology is the ability to study the activity of different molecules, and this is what can be achieved through using EBD-formed solid-state nanopores of various sizes. The nanopore-based approach allows one to study enzymes whose size exceeds that of the nanopore, since the enzyme under study must become entrapped in the nanopore. Accordingly, studying enzymes, which differ in size, requires the use of nanopores of different diameters. The time window of observation, of course, can be expanded by improving the time resolution of the ion current signal recording system, and this is the direction of the future development of nanopore-based devices. The use of devices with better time resolution will allow for further improvement of the quality of determination of enzymatic activity.

## 4. Materials and Methods

### 4.1. Chemicals

Sodium laurate (of ≥99% purity) and reduced nicotinamide adenine dinucleotide phosphate were purchased from Sigma–Aldrich (St. Louis, MO, USA). A salt mixture for preparation of phosphate-buffered saline (PBS-D) was purchased from Pierce (USA). All solutions used in our experiments were prepared using deionized ultrapure water, which was purified with a Simplicity UV system (Millipore, Molsheim, France).

### 4.2. Enzyme Solution Preparation

In the experiments reported herein, we used a 10 nM solution of the enzyme which was obtained by serial tenfold dilution of the initial 50 µM BM3 stock solution in 23 mM potassium phosphate buffer. The enzyme concentration was determined by spectrophotometry with an Agilent 8453 spectrophotometer based on a 91 mM^−1^cm^−1^ extinction coefficient for the difference in absorption at 450 nm and 490 nm [40].

### 4.3. Nanopore Fabrication

**Figure 3:**
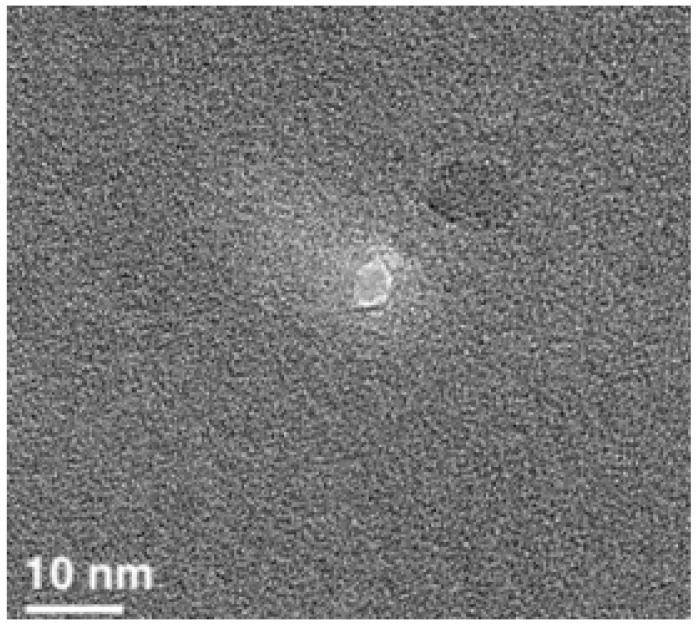
TEM image of a 5 nm nanopore formed by EBD in a 40 nm-thick SiN chip. The scale bar is 10 nm.

The nanopore was formed in a SiN chip by EBD, as described in our previous paper [33]. The size of the nanopore was smaller than the characteristic size of a BM3 molecule (12.9 × 7 × 6 nm [37]) in order to ensure its entrapment in the pore and to prevent passage of the enzyme molecules through it. Accordingly, this nanopore size amounted to 5 nm. The length of the nanopore was 40 nm. The image of the nanopore obtained by transmission electron microscopy (TEM) with a JEM 2100F electron microscope (JEOL, Ltd., Akishima, Tokyo, Japan) is shown in Figure 3.

### 4.4. Electrical Measurements

The nanopore-based detector included a measuring cell with two 700 µL chambers (cis- and trans-) separated by a SiN chip in which a 5 nm nanopore was formed. Prior to the experiment, both chambers had been filled with ultrapure water. In the experiment, the water was replaced with a 2 mM PBS-D buffer. After each measurement, the chip was washed with ultrapure water.

In the measurements, a −400 mV DC voltage was applied to the chambers with the use of Ag/AgCl electrodes. The ion current was measured within a 1000 Hz frequency band using an amplifier with an internal noise level of ~0.3 fA and recorded using a 16-bit analog-to-digital converter. The signal was processed using a Butterworth filter with a 1 kHz frequency. The detector was shielded with a Faraday cage in order to avoid external electromagnetic interference.

## 5. Conclusions

A solid-state nanopore with a diameter of ~5 nm has been formed by EBD in a 40 nm-thick silicon nitride chip. A single molecule of cytochrome P450 BM3 has been entrapped in the nanopore. No change in the ion current through the nanopore has been observed upon the entrapment of the enzyme molecule. The ion current has also been found to remain virtually unchanged after the addition of laurate to the enzyme solution. In contrast, numerous peaks on the time dependence of the ion current have occurred after the addition of an electron donor (NADPH). These peaks have indicated ion current blockades, which obviously occurred owing to fluctuations of the nanopore-entrapped enzyme molecule during its functioning. Namely, the enzyme molecule repeatedly blocked and unblocked the nanopore owing to changes in its conformation in the course of its functioning. In our experiments, this process has been observed throughout 1500 s. The results of our research can be further used in the development of highly sensitive detectors for single-molecule studies in enzymology. The results reported are also important for the determination of the physicochemical parameters of the functioning of cytochrome P450 BM3. The data reported herein can be of use for further studying of the superfamily of cytochromes P450. Furthermore, we believe that the nanopore-based approach can find its application for testing the functioning of various enzymes with different substrates, including unknown ones.

## Data Availability

Correspondence and requests for materials should be addressed to the corresponding author (Y.D.I.).
